# Targeting hepatic heparin-binding EGF-like growth factor (HB-EGF) induces anti-hyperlipidemia leading to reduction of angiotensin II-induced aneurysm development

**DOI:** 10.1371/journal.pone.0182566

**Published:** 2017-08-09

**Authors:** Seonwook Kim, Lihua Yang, Seongu Kim, Richard G. Lee, Mark J. Graham, Judith A. Berliner, Aldons J. Lusis, Lei Cai, Ryan E. Temel, Debra L. Rateri, Sangderk Lee

**Affiliations:** 1 Saha Cardiovascular Research Center at the University of Kentucky College of Medicine, Lexington, Kentucky, United States of America; 2 Cardiovascular Antisense Drug Discovery Group at the Ionis Pharmaceuticals, Inc., Carlsbad, California, United States of America; 3 Department of Medicine-Cardiology, University of California-Los Angeles School of Medicine, Los Angeles, California, United States of America; 4 Department of Pharmacology & Nutritional Sciences at the University of Kentucky College of Medicine, Lexington, Kentucky, United States of America; Ludwig-Maximilians-Universitat Munchen, GERMANY

## Abstract

**Objective:**

The upregulated expression of heparin binding EGF-like growth factor (HB-EGF) in the vessel and circulation is associated with risk of cardiovascular disease. In this study, we tested the effects of HB-EGF targeting using HB-EGF-specific antisense oligonucleotide (ASO) on the development of aortic aneurysm in a mouse aneurysm model.

**Approach and results:**

Low-density lipoprotein receptor (LDLR) deficient mice (male, 16 weeks of age) were injected with control and HB-EGF ASOs for 10 weeks. To induce aneurysm, the mice were fed a high fat diet (22% fat, 0.2% cholesterol; w/w) at 5 week point of ASO administration and infused with angiotensin II (AngII, 1,000ng/kg/min) for the last 4 weeks of ASO administration. We confirmed that the HB-EGF ASO administration significantly downregulated HB-EGF expression in multiple tissues including the liver. Importantly, the HB-EGF ASO administration significantly suppressed development of aortic aneurysms including thoracic and abdominal types. Interestingly, the HB-EGF ASO administration induced a remarkable anti-hyperlipidemic effect by suppressing very low density lipoprotein (VLDL) level in the blood. Mechanistically, the HB-EGF targeting suppressed hepatic VLDL secretion rate without changing heparin-releasable plasma triglyceride (TG) hydrolytic activity or fecal neutral cholesterol excretion rate.

**Conclusion:**

This result suggested that the HB-EGF targeting induced protection against aneurysm development through anti-hyperlipidemic effects. Suppression of hepatic VLDL production process appears to be a key mechanism for the anti-hyperlipidemic effects by the HB-EGF targeting.

## Introduction

Heparin binding EGF-like growth factor (HB-EGF), which is a member of epidermal growth factor (EGF) family member and a ligand for EGF-receptor (EGFR) [1], is involved in various pathophysiological processes including atherosclerosis and cancer development [2–6]. Among EGF family members, HB-EGF is a representative mediator for the integral EGFR transactivation by various stress conditions [7, 8]. HB-EGF regulates proliferation of vascular smooth muscle cell (VSMC) [9, 10] and inflammatory gene expression in the aortic endothelium under hyperlipidemic environment [11]. In addition, recent reports indicate that HB-EGF concentration in blood circulation correlates with circulatory cholesterol concentration [12] and risk of coronary artery disease in humans [13].

Hyperlipidemia is a key risk factor for the development of vascular diseases including aneurysm and atherosclerosis [14, 15]. For lipid or lipoprotein homeostasis, the balance of production and clearance of VLDL in the liver and capillary endothelium of peripheral tissues is critical [16]. For the production of VLDL in the liver cells, the expression and stability of apolipoprotein B (apoB) and lipid transferring protein microsomal triglyceride transfer protein (MTP) are key determinants [17]. The clearance of VLDL in circulation is mainly regulated by vascular endothelial lipoprotein lipase (LPL) [18].

Infusion of angiotensin II (AngII) into hyperlipidemic mouse models (e.g., LDLR deficient mice under high fat diet or ApoE deficient mice under chow diet) have been widely used as aneurysm models for the last decade [19, 20]. AngII infusion or hyperlipidemia alone can induce aortic aneurysm but the intensities and frequencies of aneurysm development was quite limited [14, 21]. There was a significant gender difference on aneurysm development in the model mice as male mice showed greater incidence and severity of aneurysm development [14, 22, 23].

In this study, we targeted HB-EGF gene transcription using HB-EGF-specific antisense oligonucleotide (ASO) administration to determine the targeting effects on aortic aneurysm development. In summary, we observed that the HB-EGF ASO administration induced an efficient protection against aneurysm developments in ascending and abdominal aorta. The HB-EGF targeting induced a remarkable anti-hyperlipidemic effect by suppressing hepatic VLDL secretion, which appears to be a key mechanism for the protection.

## Materials and methods

### Materials and reagent

Control ASO (549144: 5′- GGCCAATACGCCGTCA -3′) and HB-EGF ASO (597622: 5 ′-TACATTATAGTCTTGG -3′) were synthesized and purified by Ionis Pharmaceuticals as previously described [24]. The underlined text indicates cEt modified bases [25]. Poloxamer-407, a lipoprotein lipase inhibitor, was purchased from Sigma-Aldrich (Cat No. 16758). Recombinant HB-EGF (human, active form) was purchased from R&D systems (Cat No. 295-HE-CF).

### Animals

Male LDLR deficient mice (colony bred from original stock from The Jackson Laboratory; Stock No. 002207; 16 weeks of age; The strain has been backcrossed to C57BL/6J mice for 10 generations) were treated with either control or HB-EGF ASOs via intraperitoneal route at dose of 40 mg/kg/week for 10 weeks. To induce hyperlipidemia in the mice, a high fat diet (HFD; Harlan, Cat No. TD-88137) was fed *ad libitum* during the last 5 weeks. To expedite aneurysm development, AngII (Bachem, Torrance, CA) was infused at dose of 1,000 ng/kg/min via an osmotic minipump subcutaneously implanted during the last 4 weeks. All mice were maintained in an American Association for Accreditation of Laboratory Animal Care (AAALAC)-approved animal facility under protocol approved by the Institutional Animal Care and Use Committee of the University of Kentucky. http://dx.doi.org/10.17504/protocols.io.iyacfse

### *In situ* quantification of aneurysm severity and atherosclerotic lesion

Mice were perfused with saline, and hearts with attached aortas were harvested. Aortas were placed in 10% neutral buffered formalin overnight and then transferred to phosphate-buffered saline (PBS). After removal of adventitia, aortas were photographed using a digital camera (DS-Ri1; Nikon Instruments) to later measure the maximal diameter of the abdominal ascending aorta. http://dx.doi.org/10.17504/protocols.io.ix7cfrn

Then, the aortas were cut open longitudinally, pinned, and photographed *en face* with a mm unit ruler for size reference. Areas of thoracic ascending aortic intima and atherosclerotic lesions in the aortic arch area were quantified using Image Pro 7.0 software (Media Cybernetics, Bethesda, MD), as described previously [26, 27]. http://dx.doi.org/10.17504/protocols.io.izicf4e; http://dx.doi.org/10.17504/protocols.io.izfcf3n

### Noninvasive tail cuff method to measure blood pressure

Blood pressure was measured using the Kent blood pressure machine as described in the previous report [28]. Refer to S1 File Materials and Procedure-Extended for details of the procedure. http://dx.doi.org/10.17504/protocols.io.iygcftw

### Lipoprotein-associated cholesterol distribution analysis by FPLC

Blood was collected from mice in EDTA-coated tubes by cardiac puncture, and plasma was isolated by centrifugation. The cholesterol distribution among lipoprotein classes was determined after separation of plasma by gel filtration chromatography based upon the method described previously [29]. Refer to Supplementary Procedure for procedure details. http://dx.doi.org/10.17504/protocols.io.izhcf36

### Quantification of liver tissue lipid content

We followed the procedure described by Temel RE *et al*. [30]. Refer to S1 File Materials and Procedure-Extended for details. http://dx.doi.org/10.17504/protocols.io.iy7cfzn

### Hepatic VLDL secretion assay

The procedure for the assays is described in a previous report by Willecke F. *et al*. [31]. Standard C57BL/6 mice (male, 8-10weeks of age) purchased from the Jackson Laboratory (Stock No 005061) were used for the assays. Refer to S1 File Materials and Procedure-Extended for details. http://dx.doi.org/10.17504/protocols.io.izxcf7n

### Heparin-releasable plasma lipoprotein lipase (LPL) activity assays

The procedure for the assays is described in a previous report by Willecke F. *et al*. [31]. Standard C57BL/6 mice (male, 8-10weeks of age) purchased from the Jackson Laboratory (Stock No 005061) were used for the assays. Refer to S1 File Materials and Procedure-Extended for details. http://dx.doi.org/10.17504/protocols.io.iy3cfyn

### GC analysis of fecal neutral sterols

We followed the procedure described by Temel RE *et al*. [30]. Refer to S1 File Materials and Procedure-Extended for the procedure details. http://dx.doi.org/10.17504/protocols.io.iy9cfz6

### Quantitative RT-PCR

Total RNAs were extracted from mouse tissues using an RNA isolation kit (Qiagen, Cat No. 74106). cDNAs were prepared using cDNA synthesis kit (Bio-Rad, Cat No. 1708891). For amplification and quantification of PCR products, SYBR Green Master Mix (ThermoFisher Scientific; Cat No. K0223) and a PCR amplification apparatus (Bio-Rad, CFX96 Touch^™^ Real-Time PCR System) were used. Expression level of GAPDH mRNA was used for normalization. More than two sets of primers were tested for PCR amplification for each gene. Primer sequence information is described in S1 Table.

### Western blotting

Plasma samples or freshly isolated tissue samples were grinded and lysed in RIPA buffer (Cell Signaling; Cat No. 9806) containing PMSF (1mM) and protease and phosphatase inhibitor cocktails (Sigma-Aldrich; Cat No. P8340 and P5726). Protein samples were separated by SDS-PAGE using 4–20% Bio-Rad Mini-PROTEAN TGX gels following a standard procedure. Separated proteins were transferred onto polyvinylidene difluoride (PVDF) membranes using a Trans-Blot Turbo^™^ Transfer System (Bio Rad, Cat No. 1704155). After incubation with an anti-apoB antibody (Meridian Life Science, Cat No. K34005G-1; HRP-conjugated) in 1% BSA or 5% fat free milk containing TBST buffer solution, apoB bands were detected using enhanced chemiluminescent solution (Amersham, Cat No. RPN2232). ApoB bands were captured and quantified using image analysis myECL imager (Thermo Fisher, Cat No. 62236).

### Statistical analysis

Results are presented as mean ± standard deviation (SD) unless mentioned otherwise. Test group samples are compared to control group samples by Student’s T-test or Two-way ANOVA. If required, multiple comparison correction by Sidak-Bonferroni method was applied. A significant p value less than 0.05 was considered statistically different.

## Results

### HB-EGF targeting using ASO administration reduced aneurysm development

**Fig 1:**
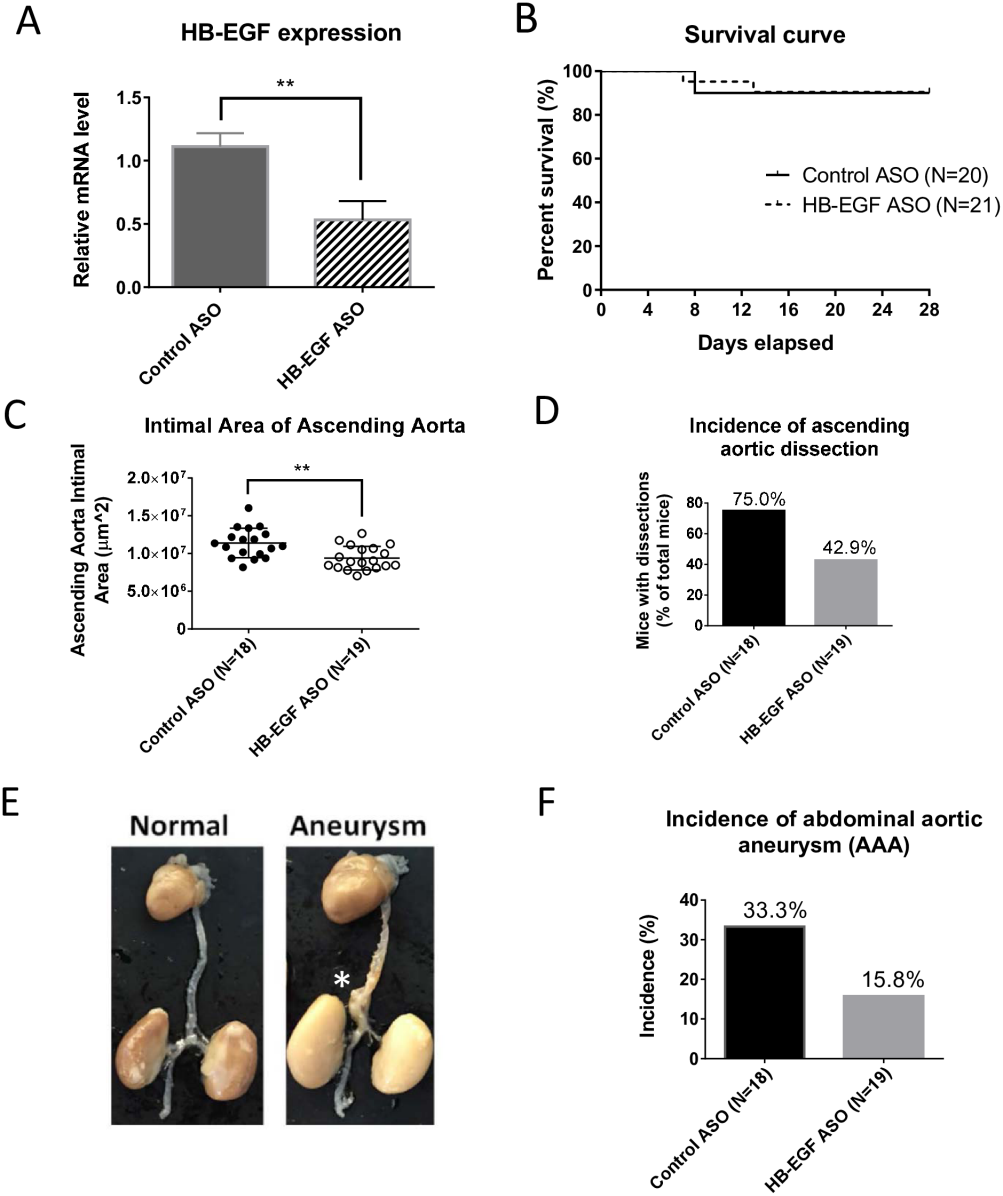
HB-EGF ASO administration significantly suppressed aneurysm formation in a mouse disease model. Male LDLR deficient mice (LDLR KO) were injected with control and HB-EGF ASOs (40 mg/kg/week) for 10 weeks (N = 20–21). The mice were fed a normal chow diet (ND) initially but changed to a high fat diet (HFD) [21% fat, 0.2% cholesterol; w/w] for the last 5 weeks of study. At the 6 week point of ASO administration, osmotic mini-pumps filled with AngII with infusion rate of 1,000ng/min/kg were implanted subcutaneously. (**A**) HB-EGF mRNA expression in the liver was determined by qRT-PCR at the termination step. (**B**) The survival curve of the model mice. (**C**) Total area of the ascending aorta intimal area. (**D**) Incidence of aortic arch dissections as percent of individuals with dissections. (**E**) Representative images of normal and aneurysmal aortas. In the aorta images, * indicates location of abdominal aortic aneurysm (AAA). (**F**) Incidence of abdominal aortic dilation. * p<0.05; ** p<0.01; and **** p<0.0001.

The HB-EGF ASO administration significantly downregulated HB-EGF mRNA expression level in liver tissue (Fig 1A). The expressions of the other EGF family members or EGFR were not affected by HB-EGF ASO administration (S1A and S1B Fig). We used male LDL deficient mice fed high fat diet (23% fat and 0.2% cholesterol; w/w) and AngII infusion for the induction of aortic aneurysm (S2A Fig for animal treatment scheme). There was no difference of body weight gains between control and HB-EGF ASO treatment groups (S2B Fig). Two mice from each group died of aortic rupture between 7 to 10 days of AngII infusion (Fig 1B).

S2C Fig shows examples of aortic arch intima of control and HB-EGF ASO treatment groups. As shown in Fig 1C, HB-EGF targeting reduced dilatation in the ascending aortic arch, suggesting a protection against thoracic aortic aneurysm (TAA) development. HB-EGF ASO administration also decreased the number of mice with dissections in the ascending aortic arch area (Fig 1D).

In addition, HB-EGF ASO administration significantly suppressed abdominal aortic aneurysm (AAA) development. Representative intact aorta images with and without AAA were shown in Fig 1E. Images for all aortas of control and HB-EGF ASO treatment groups were listed in S3A and S3B Fig. There was a significant reduction of the number of mice with abdominal aortic dilation incidence by HB-EGF targeting (Fig 1E and S3C Fig). We also confirmed that HB-EGF ASO administration effectively suppressed atherosclerotic lesion formation in the aortic arch lumen area as shown in S3D Fig.

Other groups previously reported that prenatal whole body- or vascular endothelial-specific HB-EGF gene deletions induced cardiac hypertrophy with gross enlargement of heart ventricular chambers [32–34]; however, postnatal induction of HB-EGF gene deletion did not induce the phenotype change [35]. In a control experiment using LDLR deficient mice under normal diet, we tested the effects of HB-EGF ASO administration on the cardiac structure. First, we confirmed downregulation of HB-EGF mRNA level by the HB-EGF ASO administration in multiple tissues including liver, aorta, and kidney (S4A Fig). There was no development of vascular defects like aneurysm under the diet condition (S4B Fig). The HB-EGF ASO administration did not induce cardiac hypertrophy (S4C and S4D Fig) or changes of ventricular chamber size and morphology of heart muscle tissues (S5A and S5B Fig). Interestingly, we detected a significant reduction of basal blood pressure by the HB-EGF ASO administration without change of heart rate (S5C and S5D Fig). The HB-EGF ASO administration still induced a significant suppression of circulatory lipid levels in the LDLR deficient mice (S5E and S5F Fig).

Separately, we tested the effects of HB-EGF ASO administration in wild type C57BL/6 mice under normal diet. The C57BL/6 was the genetic background of the LDLR deficient mice that we adopted for aneurysm study. As expected, the HB-EGF ASO administration did not induce any vascular defects like aneurysm or atherosclerosis (data not shown). There were no changes of heart size or chamber structures by the ASO administration (data not shown). However, still we observed a significant reduction of circulatory lipid levels by the HB-EGF ASO administration in the C57BL/6 mice (S6A and S6B Fig).

### HB-EGF targeting induced anti-hyperlipidemic effects by suppressing the hepatic VLDL secretion

**Fig 2:**
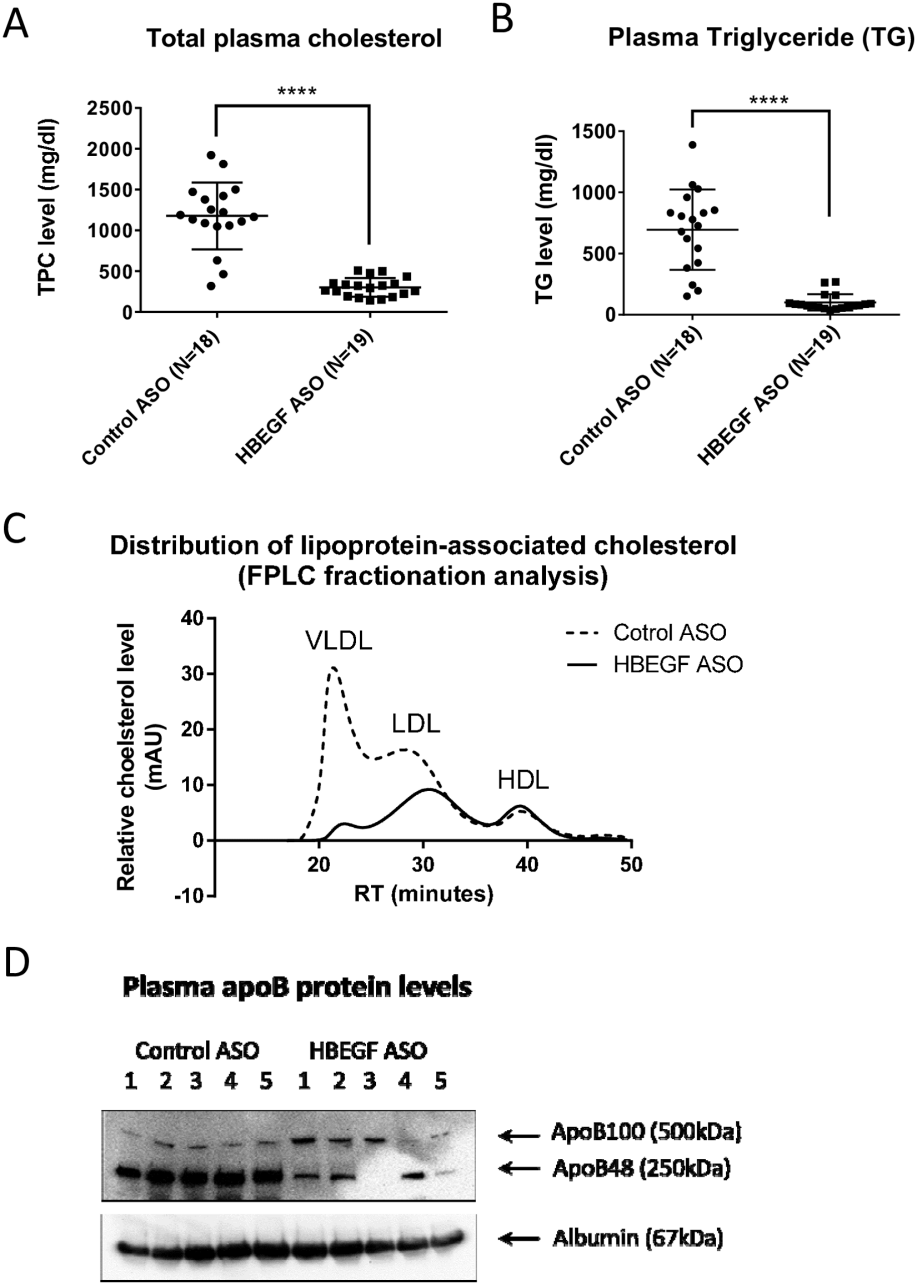
HB-EGF ASO administration suppressed circulatory lipid concentrations. Refer to Fig 1 legend for the LDLR deficient mice treatment. (**A-B**) Plasma total cholesterol and triglyceride (TG) concentrations in the plasma samples collected at the termination step. (**C**) FPLC fractionation analysis for the lipoprotein-associated cholesterol in the plasma. Four plasma samples, chosen from the median range of cholesterol concentration of each group, were pooled for the FPLC analysis. (**D**) ApoB and albumin levels in the plasma samples from the median range of cholesterol concentration of each group were compared by western blotting analysis (N = 5). **** p<0.0001.

HB-EGF ASO induced a remarkable suppression of systemic total cholesterol and TG concentrations in the LDLR deficient mice under HFD (Fig 2A and 2B). FPLC fractionation of lipoprotein-associated cholesterol of plasmas collected at termination step showed remarkable and moderate downregulations of VLDL and LDL-cholesterols in circulation, respectively (Fig 2C); however, there was no change for HDL-associated cholesterol concentration by the HB-EGF ASO administration. In correspondence, there was a downregulation of apoB protein concentration in blood circulation (Fig 2D).

**Fig 3:**
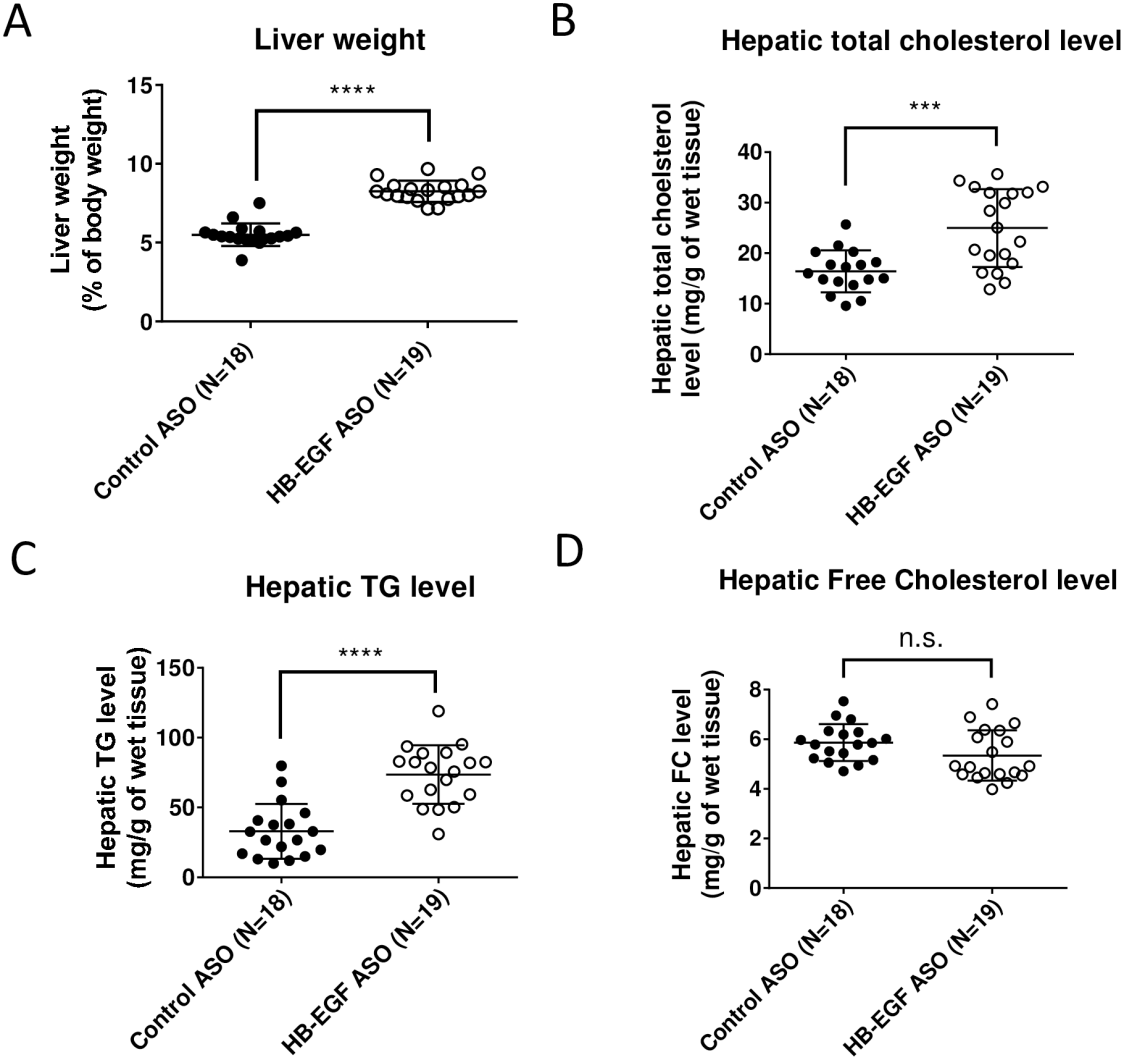
HB-EGF ASO administration increased neutral lipid contents in liver. Refer to Fig 1 legend for animal treatment. (**A**) At the termination step, liver weight was measured as a percent of the total body weight. (**B-D**) Concentrations of TG, total cholesterol, and free cholesterol in the liver tissues were quantified. * p < 0.05; *** p<0.001; **** p<0.0001; and n.s. = not significant.

We observed a significant increase of liver weight (Fig 3A) and elevation of hepatic neutral lipid contents (i.e., TG and cholesterol ester) by the HB-EGF ASO treatment (Fig 3B and 3C). There was no difference of free cholesterol content in the liver (Fig 3D).

**Fig 4:**
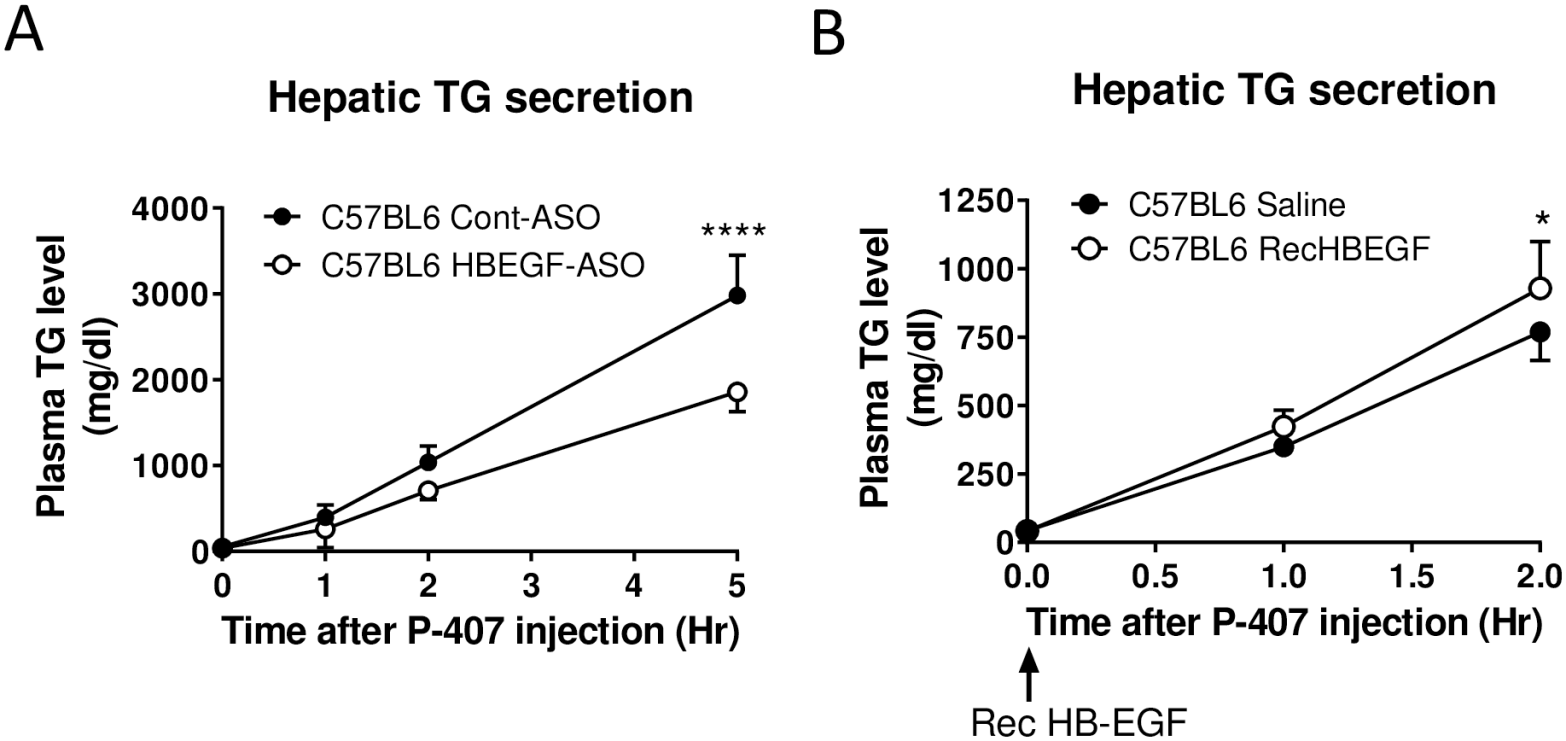
HB-EGF targeting using ASO administration induced suppression of hepatic VLDL-associated TG secretion rate. (**A**) C57BL/6 mice (male, 10 weeks of age) were pretreated with control and HB-EGF ASOs for 6 weeks (40 mg/kg/week). For the secretion assay, a lipoprotein lipase inhibitor poloxamer-407 (P-407) (1.0 g/kg of body weight) was injected intraperitoneally and he changes of TG levels in the plasma were determined for 0–5 hours (N = 3–5 per group). (**B**) C57BL/6 mice (male, 10 weeks of age) were tail-vein injected with recombinant HB-EGF (2 mg/kg of body weight) at 0 hour point of P-407 injection. The TG levels in the plasma samples for 0–2 hour points were determined (N = 5 per group). * p<0.05; and **** p<0.0001.

The circulatory VLDL level was suppressed but there was simultaneous elevation of neutral lipid contents in the liver by the HB-EGF administration. Thus, we hypothesized that HB-EGF positively regulates VLDL secretion from the liver. To test this hypothesis, we directly measured hepatic VLDL secretion rate using C57BL/6 mice [31]. As shown in Fig 4A, the HB-EGF ASO administration induced a significant suppression of hepatic VLDL secretion rate; in contrast, injection of recombinant HB-EGF (human, active form) significantly increased hepatic VLDL secretion rate although the rate increase was transient for 1–2 hours. A group previously demonstrated that human HB-EGF was functional in mouse system as shown in the mouse system with humanized HB-EGF [36].

Delayed clearance of TG rich VLDL particles from the circulation by vascular endothelial lipoprotein lipase (LPL) can lead to elevation of circulatory VLDL level [18, 37]. However, there were no changes of heparin-releasable plasma TG hydrolytic activity by 6 weeks of HB-EGF ASO administration or by the recombinant HB-EGF injection (S7A and S7B Fig). Suppression of intestinal cholesterol absorption and consequently increasing fecal neutral sterol excretion has been shown to decrease circulating atherogenic apoB-containing lipoproteins [38]; however, we confirmed that there was no change of fecal neutral sterol excretion rate by the HB-EGF ASO administration (S7C Fig).

## Discussion

In this study, we demonstrated that the HB-EGF targeting significantly suppressed aneurysm and atherosclerotic lesion development in a mouse disease model. Mechanistically, an efficient lipid-lowering by the HB-EGF targeting appears to be a primary mechanism for the protection against the aneurysm and atherosclerosis. This study also indicates that the HB-EGF via EGFR signaling could be an important positive regulator for the production of VLDL in liver. The injection of recombinant HB-EGF enhanced hepatic VLDL production rate, but the HB-EGF ASO administration suppressed circulatory lipid levels in both normolipidemic and hyperlipidemic conditions, which suggested that HB-EGF would be a general regulator for the regulation of hepatic VLDL production. This new information could be of potential importance in understanding lipoprotein metabolism and lipid homeostasis in the liver and blood circulation [37, 39].

The molecular and cellular mechanism for the regulation by HB-EGF signaling on hepatic VLDL production is unclear yet. The process of VLDL production in the hepatocytes is highly complicated process, which is controlled by multi-factors including lipid substrate availability [17, 40] and expression and function of apoB and microsomal triglyceride transfer protein (MTP) proteins in the ER lumen space [41, 42]. Recently, Lee RG et al. showed that suppressions of apoB or MTP expressions in the liver tissue using ASO administrations induced efficient reductions of circulating lipid levels but with significant increases of neutral lipid levels in the liver tissue [25]. Because the HB-EGF ASO administration did not induce significant changes of apoB mRNA or MTP expression levels (mRNA and protein) in the liver (data not shown), the apoB- or MTP-independent mechanism might be involved in suppressing hepatic VLDL production by the ASO administration. The maturation of primordial VLDL initially synthesized in endoplasmic reticulum (ER) lumen space and subsequent secretory process in the Golgi apparatus are another important factors [43]. Because there was a significant elevation of neutral lipid contents in the liver and increase of lipid droplet formation in the liver cell, the HB-EGF targeting appears to cause delay of mobilization of lipid substrates from the cytosolic lipid droplets in the hepatocytes [40]. Other groups showed that the combination of suppression of apoB expression and enhanced *de novo* lipogenesis, which happens under insulin resistance or metabolic syndrome conditions, induced enlargement of VLDL particles in circulation [44]. Because we observed that both TG and apoB levels were reduced by the HB-EGF ASO administration, the size of the VLDL particle appears to be constant. Thus, not the deficiency of apoB or MTP but the insufficiency of the lipid substrate for VLDL assembly appears to be a key mechanism for the suppression of VLDL production in the liver by the HB-EGF ASO administration.

Previous reports delineated that local expression of HB-EGF in the aortic vessel was associated with burden of atherosclerosis [2, 4, 45]. Though the liver tissue is one of the top organs with efficient ASO distribution [46], the HB-EGF ASO administration significantly suppressed HB-EGF expression in the aorta, which may contribute to the protection against development of aneurysm and atherosclerosis. We showed that the HB-EGF-EGFR signaling mediates inflammatory gene transcription in the vascular endothelial cells by oxidized phospholipids [11] and the signaling also regulates the proliferation and migration of the vascular smooth muscle cells [10]. An additional complication is that HB-EGF mediates angiotensin II signaling in the vascular smooth muscle cells [10, 47–49]. Thus, local suppression of HB-EGF expression may directly inhibit angiotensin II signaling in the vessel wall. It would be difficult to study tissue-specific effects of the HB-EGF targeting using ASO administration. Further use of inducible VSMC or vascular endothelial-specific HB-EGF gene knockout systems would be helpful to define the local effects of the HB-EGF knockdown.

Two separate groups developed HB-EGF floxed mouse system for the induction of tissue-specific HB-EGF knockout using loxP-Cre system [32, 33]. Prenatal whole body-, vascular endothelium-, or vascular smooth muscle cell-specific knockouts of HB-EGF induced cardiac hypertrophy with severe defects of valvulogenesis causing gross enlargement of ventricular chambers [32–34]. Similar phenotype was shown by genetic knockouts of EGFR or TACE/ADAM17, which is a downstream receptor of HB-EGF and the metalloproteinase that activates HB-EGF on cell surface, respectively. Mice with defects of HB-EGF ectodomain shedding fragment also showed similar cardiac hypertrophy [50]. In contrast, post-natal induction of HB-EGF gene deletion in the vascular endothelium or in the liver did not show structural defects of heart [35, 51, 52]. We also show that the HB-EGF ASO administration does not cause structural problems in the heart. Compared with genetically modified model systems, the suppression of HB-EG expression by HB-EGF ASO was moderate, about 50% mRNA level reduction. In control experiments using LDLR deficient mice under normal diet or C57BL/6 mice under normal diet, we confirmed absence of deleterious effects of HB-EGF ASO on the heart structure and size of ventricular chambers.

Takemura et al. showed that the induction of liver-specific HB-EGF knockdown enhanced liver injuries induced by thioacetamide (TAA), CCl_4_, or bile duct ligation procedure [51–53]. The mechanism for the increase of sensitivity to the liver damages is not clear yet. Possibly, the upregulation of neutral lipid contents in the liver might be one cause for the increased liver damage. The lipid accumulation in the liver by HB-EGF ASO administration suggests possible limitation in applying the ASO for the subjects with hyperlipidemia or hyperlipidemia-associated vascular diseases. Lee RG et al. showed that the administration of apoB and MTP ASOs in the liver induced increases of neutral lipid contents in the liver in the LDLR deficient mice under HFD [25]. However, the apoB ASO induced less accumulation of lipids with formation of smaller size of lipid droplets in the liver. Recently, Conlon et al. showed the mechanism for the differences of lipid accumulation by apoB and MTP ASOs [54]. The apoB ASO induced an autophagic pathway that helps removal of neutral lipids in the liver cells. Because the lipid disposing autophagic pathway requires MTP function, MTP targeting by MTP ASO causes severe fat accumulation in the liver. The HB-EGF ASO administration keeps MTP expression intact in the liver, which may be a favorable sign for the induction of the lipid disposing autophagic pathway.

Interestingly, we observed that the HB-EGF ASO administration induced a significant downregulation of basal blood pressure in control experiments using LDLR deficient mice as shown in S5C Fig. The mechanism for the anti-hypertensive effects is unclear yet. Previous reports on the effects of whole-body knockout of HB-EGF on the blood pressure were inconsistent [32, 33, 55]; two groups showed that the HB-EGF knockouts did not change basal blood pressure [32, 55], but one group showed a significant lowering of blood pressure [33]. We presume that the reduction of blood pressure was not by direct effects of heart dysfunction because the ASO administration did not induce apparent changes of heart structure. As one clue, we detected that the HB-EGF ASO administration significantly suppressed HB-EGF expression in the kidney. Previously, other groups reported that targeting of HB-EGF induced protections against vasospatic response by endothelin-1 administration [55] or against renal injuries induced by chronic infusion of AngII [35] and ischemic reperfusion [56]. Vascular endothelial-specific HB-EGF knockout also leads to protection against development of progressive crescentic glomerulonephritis [57]. For determination of the mechanism of the anti-hypertensive effects shown by the HB-EGF ASO administration, further study of the effects on the kidney function would be required.

Previous reports showed that an EGFR blocker gefitinib was shown to downregulate circulatory lipid levels in a mouse model [58] and another EGFR blocker AG1478 also induced a significant protection against atherosclerotic lesion development in a hyperlipidemic mouse model [59]. Multiple EGFR ligands are expressed in the liver as shown in S1A and S1B Fig. Previous reports suggested that each EGF family members induces both overlapped and distinctive intracellular EGFR signaling depending on the type of receptor it interacts with [60, 61]. Although this study was focused on HB-EGF signaling, the results of this study suggests a possibility that the other EGF family members than HB-EGF might also contribute to the pathophysiological regulation of hepatic VLDL production, which would be an important subject in understanding homeostasis of hepatic lipoprotein metabolism.

HB-EGF is involved in the development and advancement of multiple types of cancer [5, 36, 62]. Targeting of HB-EGF has been aimed to inhibit growth and metastasis of various cancers [36, 63–72]. Diverse approaches for HB-EGF targeting have been tested including blocking antibodies [63, 65, 66, 70] and inert diphtheria toxin derivatives [71, 73]. Unexpectedly, the HB-EGF blocking antibodies caused neurological side effects, which caused discontinuation of human trial [70]. Similar neurological effect was also shown in mice with brain-specific HB-EGF knockout [74]. Because ASO does not pass through blood brain barrier, we expect absence or far less brain-associated side effects in applying the ASO for human system [46].

We observed that the HB-EGF ASO administration protects against both thoracic and abdominal aortic aneurysms (TAA and AAA). Multiple groups demonstrated that TAA and AAA have differential pathological mechanisms [14, 15]; however, hyperlipidemia appears to be a common risk factor for both types of aneurysms. Collectively, in this study, we demonstrated HB-EGF targeting significantly reduced aortic aneurysm developments in the aorta. The HB-EGF targeting remarkably suppressed circulating lipid concentration by delaying hepatic VLDL production. Further evaluation of liver- or brain-associated side effects would be required to evaluate the usefulness of HB-EGF targeting against aneurysm and possibly against atherosclerosis.

## Supporting information

S1 Fig HB-EGF ASO administration downregulated hepatic HB-EGF mRNA levels. (**A**) LDLR deficient mice (LDLR KO) fed normal diet (ND) were injected weekly with control and HB-EGF ASOs for 12 weeks (40 and 20 mg/kg/wk by 6 week interval). Relative mRNA levels of the EGF family members, EGFR, and ERBB4 in the liver tissues were determined by RT-PCR. The number in () indicates PCR cycle no. The cycle number for each gene was optimized to detect differences of template amounts by standard reactions using serial dilution of pooled RNA samples. PCR reactions for amphiregulin, which is a member of EGF family, with 2 different sets of primers showed no products. Housekeeping gene GAPDH product bands were used for normalization. Similar results were reproduced by more than 2 times of repeated PCR reactions using the same total RNA samples. **(B)** Quantification of the band intensities image analysis software program. * p < 0.05.
(TIF)

S2 Fig HB-EGF ASO administration effectively suppressed thoracic aortic aneurysm (TAA) formation. (**A**) Experimental design for the induction of aneurysm in male LDLR deficient mice. Male LDLR deficient mice were injected weekly intraperitoneally with either control or HB-EGF ASOs (40 mg/kg/wk) for 10 weeks (N = 20–21). The mice were fed normal diet (ND) initially but changed to a high fat diet (HFD) [21% fat, 0.2% cholesterol (w/w)] for the last 5 weeks of the study. At the 6 week point, osmotic mini-pumps were filled with AngII (1,000 ng/min/kg) and implanted subcutaneously. (**B**) Weekly body weight changes of the disease model mice. Starting points for HFD feeding and AngII infusion are marked with arrows. Values are mean plus standard deviation (SD). (**C**) Representative examples of aortic arch intimal images for the control and HB-EGF ASO groups. The ‘a’ indicates location of aortic dissection; and ‘b’ indicates lesion area covered with plaque accumulation in subendothelial space. The intimal perimeter of the ascending aorta was traced in the right panel image. Scale bars inserted have units of mm.
(TIF)

S3 Fig HB-EGF ASO administration suppressed abdominal aortic aneurysm (AAA) and atherosclerotic lesion formation. Refer to S2A Fig for experimental design scheme. (**A-B**) Images of aortas for control and HB-EGF ASO treatment groups. * indicates AAA located at the suprarenal area of the abdominal aorta. (**C**) At termination, the maximal diameter of the suprarenal abdominal aorta was measured. (**D**) *En face* measurement of aortic arch intimal atherosclerotic lesion area as a percent of total aortic arch lumen area.
(TIF)

S4 Fig The effects of HB-EGF ASO administration in LDLR deficient mice under normal diet condition. Male LDLR deficient mice were injected weekly intraperitoneally with either control or HB-EGF ASOs (40 mg/kg/wk) for 6 weeks (N = 5 per group). The mice were fed normal standard diet. There was no treatment of AngII in the mice (as non-disease control mice). (**A**) At the termination step, liver, aorta, heart, and kidney tissues were isolated for the measurement of HB-EGF expression levels by qRT-PCR analyses. (**B**) After removing adventitia from the aortic structure, the diameters of aortic arch and suprarenal area were measured. (**C**) Alignment of images of intact aortas linked with heart and kidney tissues. (**D**) The size of heart and kidney was measured for long and short dimensions of tissues.
(TIF)

S5 Fig The effects of HB-EGF ASO administration on the heart structure and blood pressure in LDLR deficient mice under normal diet. Refer to S4A Fig for the mouse treatment. (**A**) The representative images of heart sections (**B**) Morphology of the heart muscle cells (x 200) (**C, D**) Systolic blood pressure and heart rate as measured by tail-cuff method as described in the Procedure section. (**E, F**) At the termination step, plasma samples were collected by heart puncture. The levels of total cholesterol and TG in the plasmas were quantified.
(TIF)

S6 Fig The effects of HB-EGF ASO administration on the in C57BL/6 mice under normal diet. C57BL/6 mice (male, 10 weeks of age) were injected weekly intraperitoneally with either control or HB-EGF ASOs (40 mg/kg/wk) for 6 weeks (N = 5 per group). The mice were fed normal standard diet. There was no treatment of AngII (as non-disease wild type control mice) (**A, B**) At the termination step, the plasma samples of each animal were collected by heart puncture bleeding. The levels of plasma total cholesterol and TG were quantified.
(TIF)

S7 Fig HB-EGF is not involved in heparin-releasable TG hydrolytic activities or regulating fecal neutral sterol excretion rate. (**A**) Heparin-releasable plasma TG hydrolytic activities were measured in C57BL/6 mice, which is genetic background of LDLR KO mice, after 3 weeks of control or HB-EGF ASO administrations (50 mg/kg/wk) (N = 5). Downregulation of hepatic HB-EGF expression levels by the HB-EGF ASO administration was separately confirmed by qRT-PCR. (**B**) Heparin-releasable plasma TG hydrolytic activities were measured in C57BL/6 mice (male, 10 weeks of age) after one time tail-vein injection of either saline or recombinant HB-EGF (2 mg/kg of body weight; human active form) at 2 hours before heparin injection. (N = 5) (**C**). The HB-EGF ASO administration for 6 weeks (40 and 20 mg/kg/wk for 4 and 2 weeks consequently) in LDLR deficient mice under normal diet did not change fecal neutral sterol excretion rate. (N = 5) Refer to Supplemental Procedure-Extended for the procedure details. n.s. = not significant.
(TIF)

S1 Table Primer sequence information used for PCR reactions in the study. (PDF)

S1 File Materials and Procedure-Extended. (PDF)

S2 File ARRIVE guideline. (PDF)

## Abbreviations

AAA: abdominal aortic aneurysm
AngII: angiotensin II
ApoB: apolipoprotein B
ASO: antisense oligonucleotide
EGF: epidermal growth factor
EGFR: EGF receptor
HB-EGF: heparin-binding EGF-like growth factor
HFD: high fat diet
LDL: low-density lipoprotein
LDLR: LDL receptor
MTP: microsomal triglyceride transfer protein
TAA: thoracic aortic aneurysm
TG: triglyceride
VLDL: very low-density lipoprotein
VSMC: vascular smooth muscle cell
