# Supplementary figures and images for: Targeting hepatic heparin-binding EGF-like growth factor (HB-EGF) induces anti-hyperlipidemia leading to reduction of angiotensin II-induced aneurysm development
Source: PLoS One. 2017 Aug 9;12(8):e0182566. doi: 10.1371/journal.pone.0182566 (PMC5549937; doi:10.1371/journal.pone.0182566)

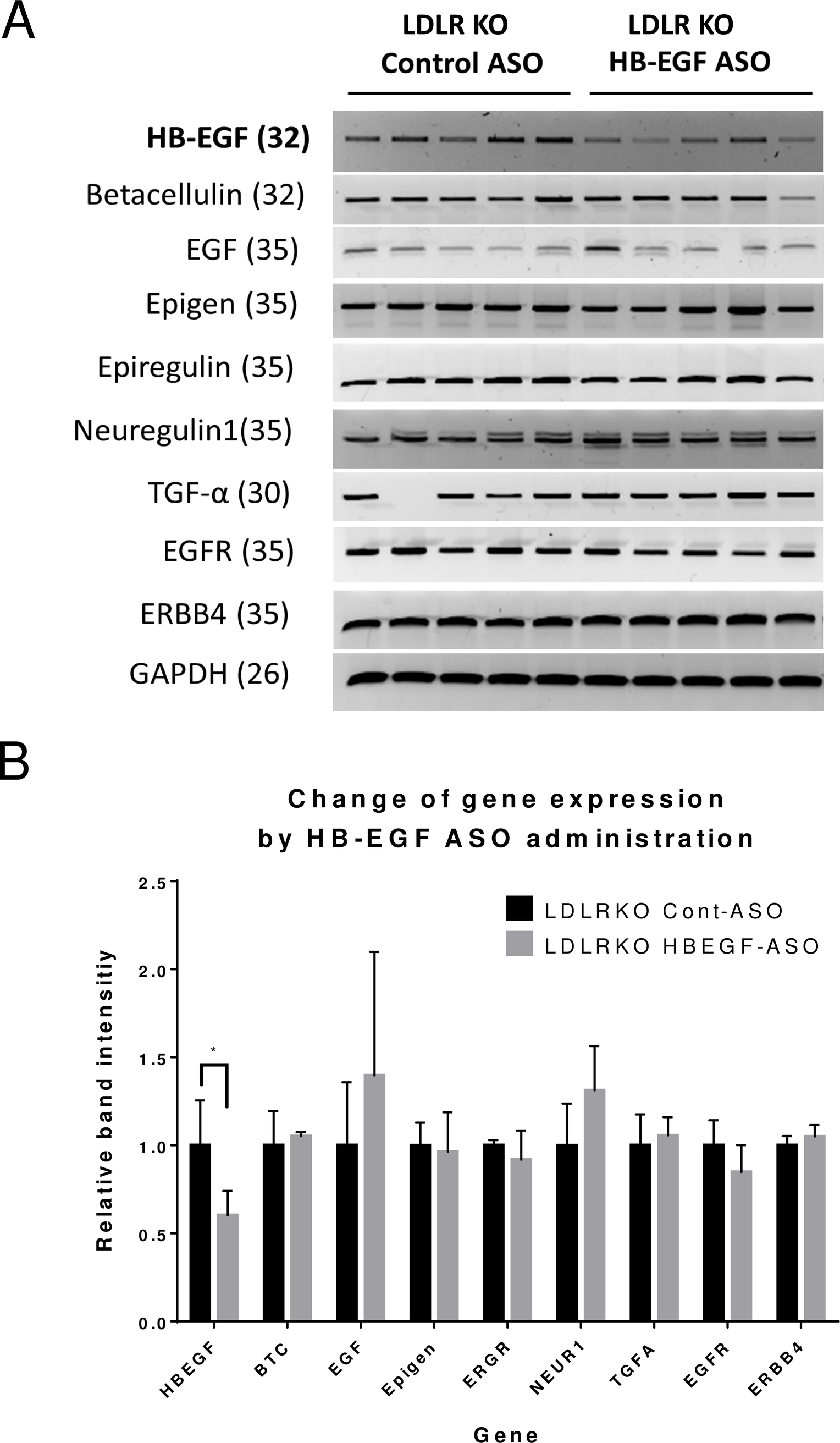

Supplement: S1 Fig — (A) LDLR deficient mice (LDLR KO) fed normal diet (ND) were injected weekly with control and HB-EGF ASOs for 12 weeks (40 and 20 mg/kg/wk by 6 week interval). Relative mRNA levels of the EGF family members, EGFR, and ERBB4 in the liver tissues were determined by RT-PCR. The number in () indicates PCR cycle no. The cycle number for each gene was optimized to detect differences of template amounts by standard reactions using serial dilution of pooled RNA samples. PCR reactions for amphiregulin, which is a member of EGF family, with 2 different sets of primers showed no products. Housekeeping gene GAPDH product bands were used for normalization. Similar results were reproduced by more than 2 times of repeated PCR reactions using the same total RNA samples. (B) Quantification of the band intensities image analysis software program. * p < 0.05. (TIF) [file pone.0182566.s001.tif]

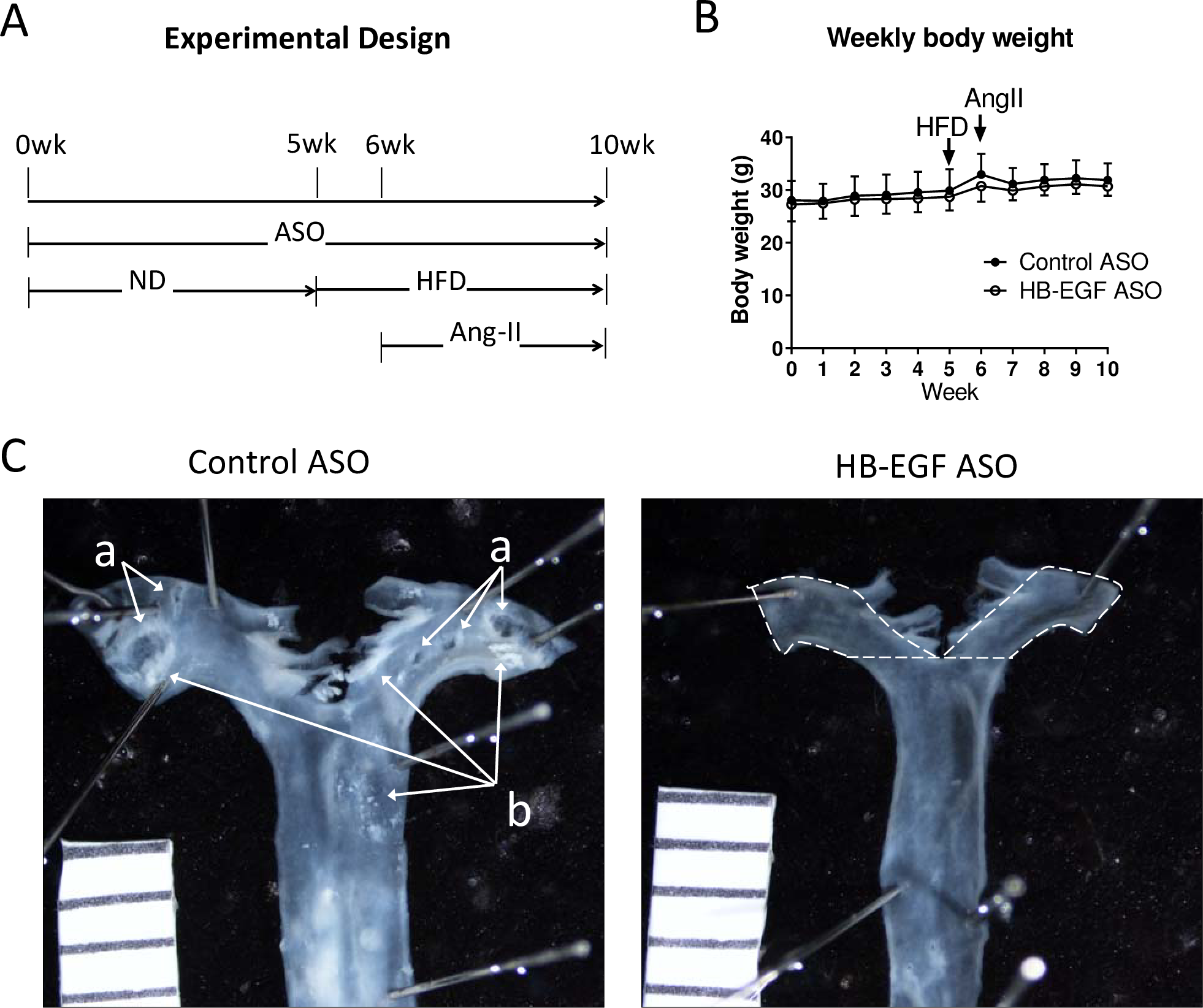

Supplement: S2 Fig — (A) Experimental design for the induction of aneurysm in male LDLR deficient mice. Male LDLR deficient mice were injected weekly intraperitoneally with either control or HB-EGF ASOs (40 mg/kg/wk) for 10 weeks (N = 20–21). The mice were fed normal diet (ND) initially but changed to a high fat diet (HFD) [21% fat, 0.2% cholesterol (w/w)] for the last 5 weeks of the study. At the 6 week point, osmotic mini-pumps were filled with AngII (1,000 ng/min/kg) and implanted subcutaneously. (B) Weekly body weight changes of the disease model mice. Starting points for HFD feeding and AngII infusion are marked with arrows. Values are mean plus standard deviation (SD). (C) Representative examples of aortic arch intimal images for the control and HB-EGF ASO groups. The ‘a’ indicates location of aortic dissection; and ‘b’ indicates lesion area covered with plaque accumulation in subendothelial space. The intimal perimeter of the ascending aorta was traced in the right panel image. Scale bars inserted have units of mm. (TIF) [file pone.0182566.s002.tif]

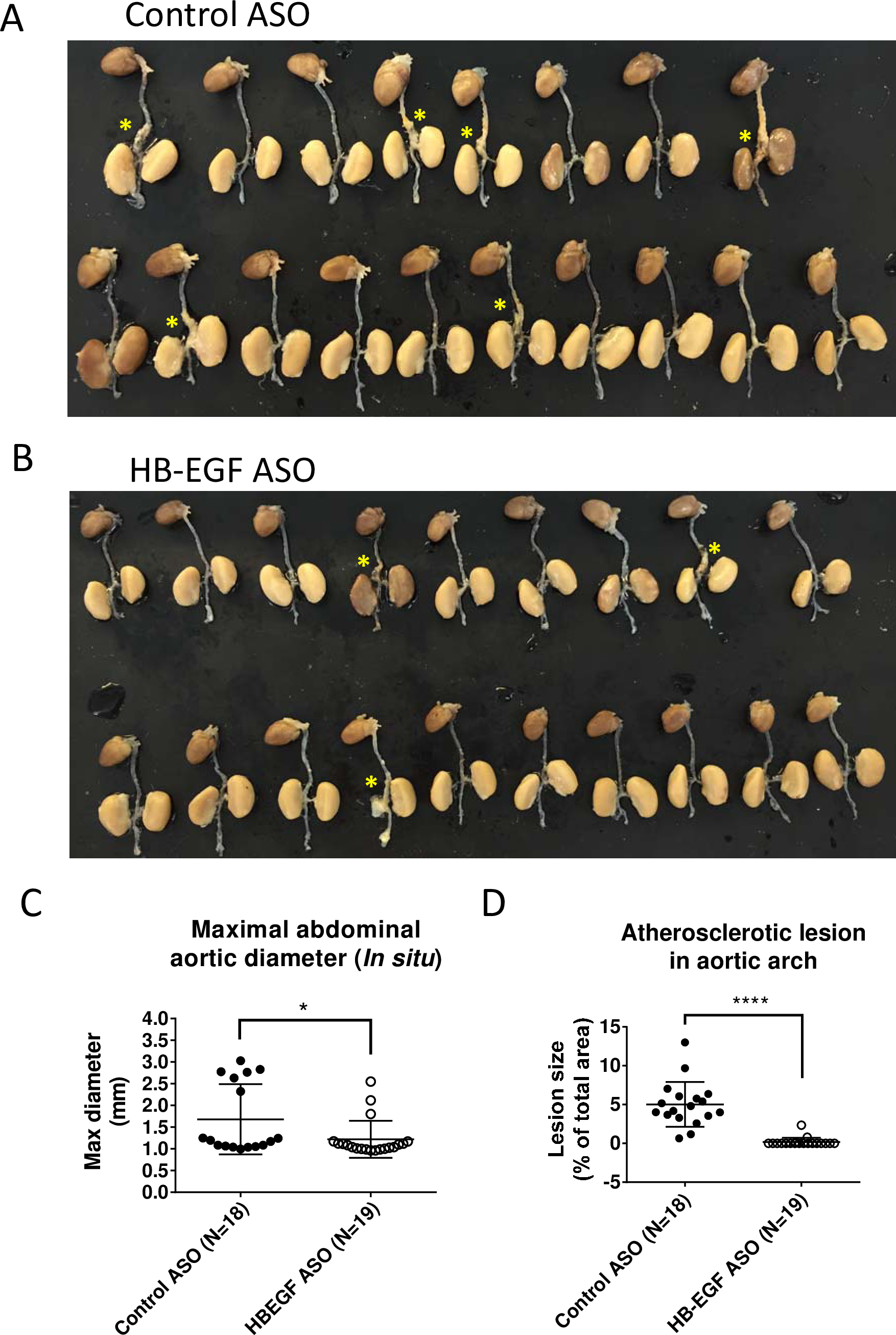

Supplement: S3 Fig — Refer to S2A Fig for experimental design scheme. (A-B) Images of aortas for control and HB-EGF ASO treatment groups. * indicates AAA located at the suprarenal area of the abdominal aorta. (C) At termination, the maximal diameter of the suprarenal abdominal aorta was measured. (D) En face measurement of aortic arch intimal atherosclerotic lesion area as a percent of total aortic arch lumen area. (TIF) [file pone.0182566.s003.tif]

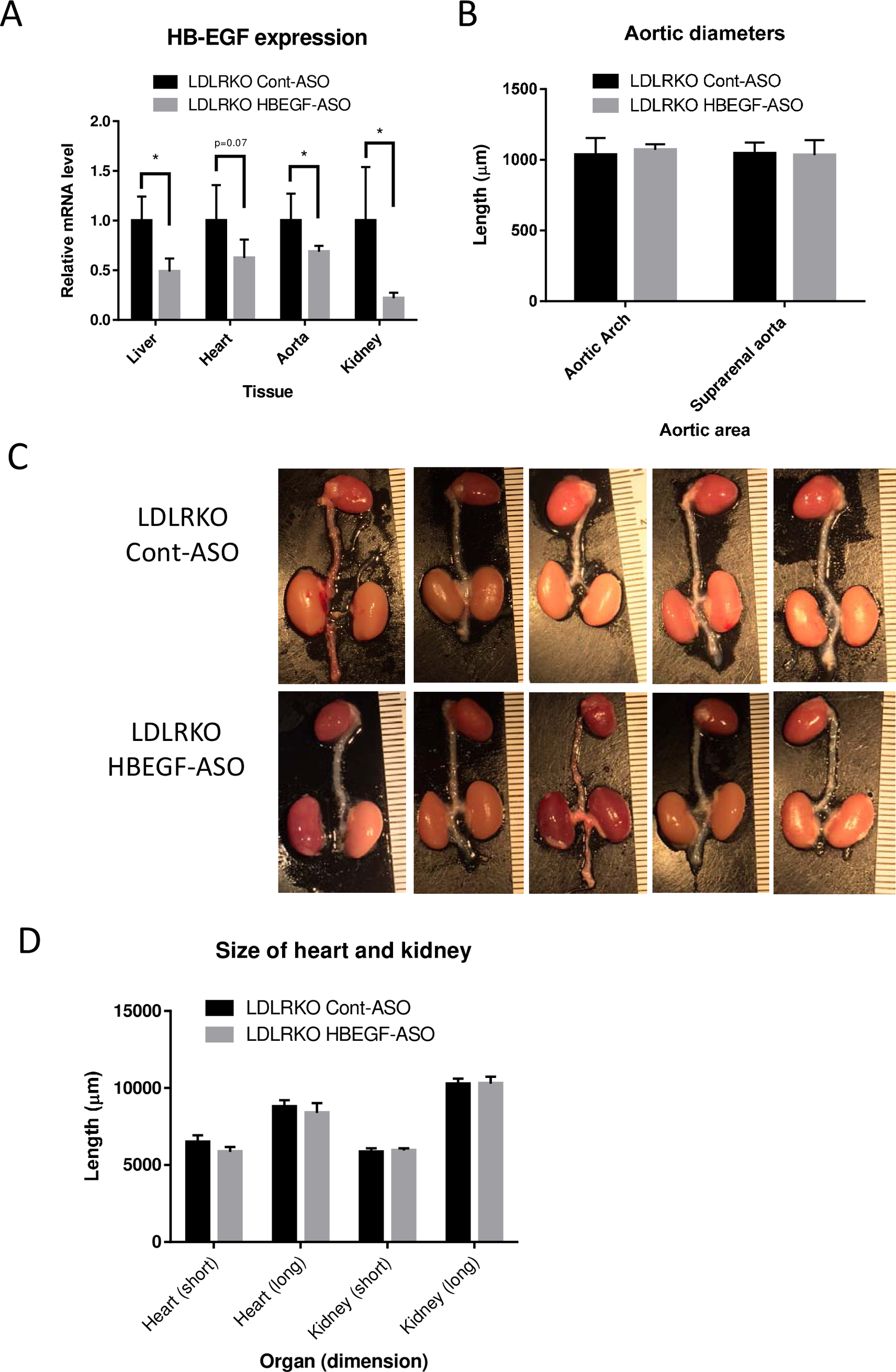

Supplement: S4 Fig — Male LDLR deficient mice were injected weekly intraperitoneally with either control or HB-EGF ASOs (40 mg/kg/wk) for 6 weeks (N = 5 per group). The mice were fed normal standard diet. There was no treatment of AngII in the mice (as non-disease control mice). (A) At the termination step, liver, aorta, heart, and kidney tissues were isolated for the measurement of HB-EGF expression levels by qRT-PCR analyses. (B) After removing adventitia from the aortic structure, the diameters of aortic arch and suprarenal area were measured. (C) Alignment of images of intact aortas linked with heart and kidney tissues. (D) The size of heart and kidney was measured for long and short dimensions of tissues. (TIF) [file pone.0182566.s004.tif]

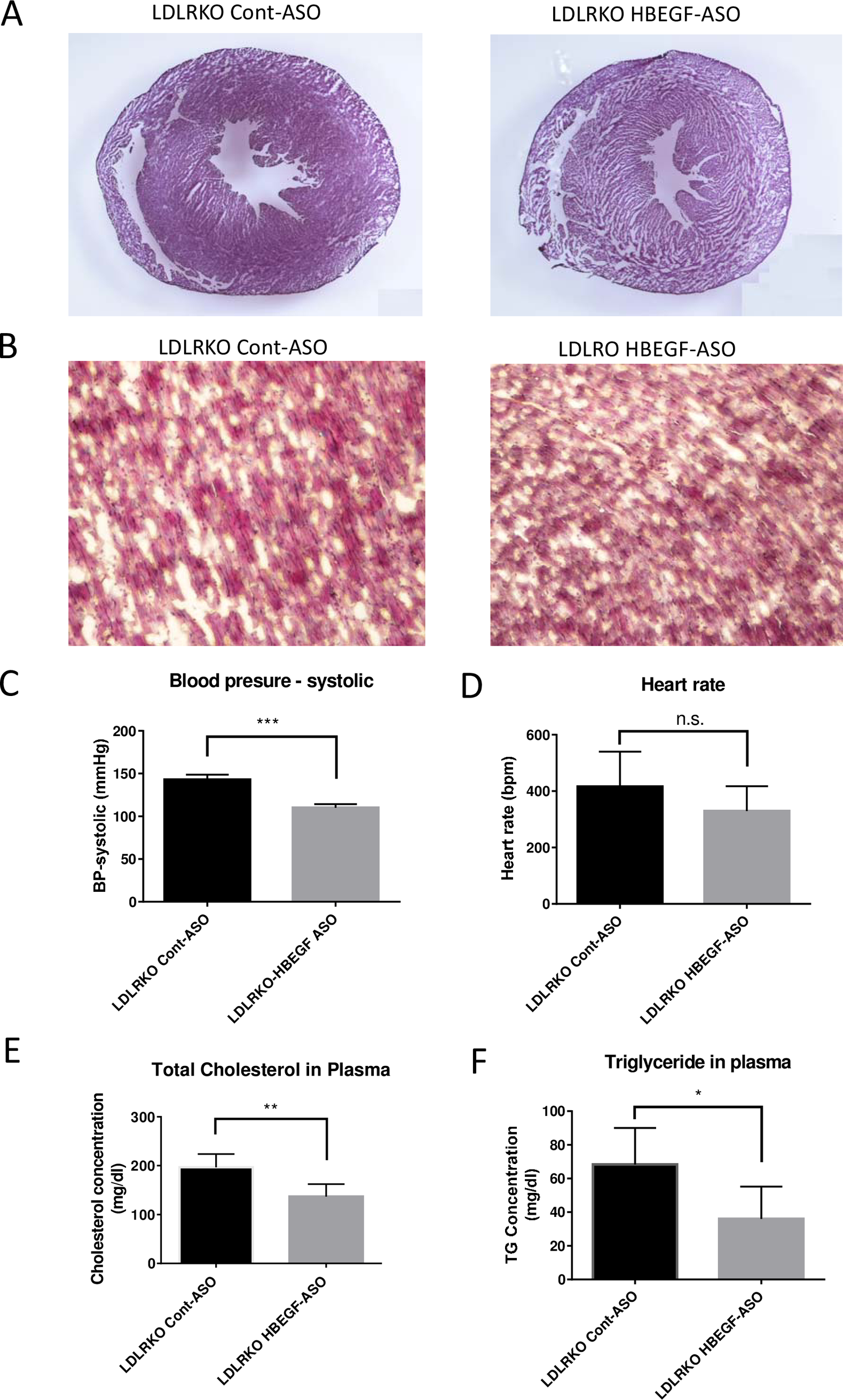

Supplement: S5 Fig — Refer to S4A Fig for the mouse treatment. (A) The representative images of heart sections (B) Morphology of the heart muscle cells (x 200) (C, D) Systolic blood pressure and heart rate as measured by tail-cuff method as described in the Procedure section. (E, F) At the termination step, plasma samples were collected by heart puncture. The levels of total cholesterol and TG in the plasmas were quantified. (TIF) [file pone.0182566.s005.tif]

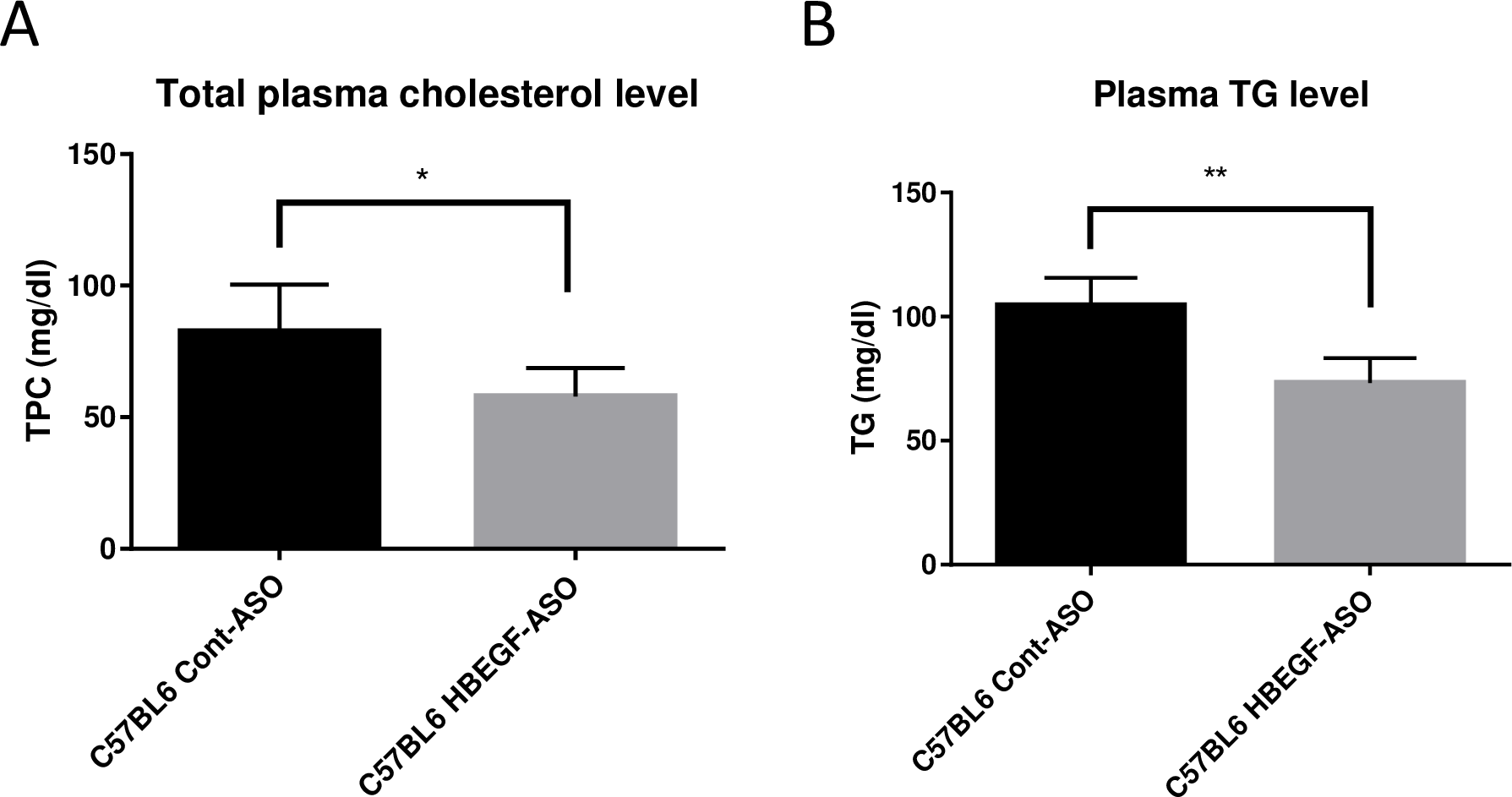

Supplement: S6 Fig — C57BL/6 mice (male, 10 weeks of age) were injected weekly intraperitoneally with either control or HB-EGF ASOs (40 mg/kg/wk) for 6 weeks (N = 5 per group). The mice were fed normal standard diet. There was no treatment of AngII (as non-disease wild type control mice) (A, B) At the termination step, the plasma samples of each animal were collected by heart puncture bleeding. The levels of plasma total cholesterol and TG were quantified. (TIF) [file pone.0182566.s006.tif]

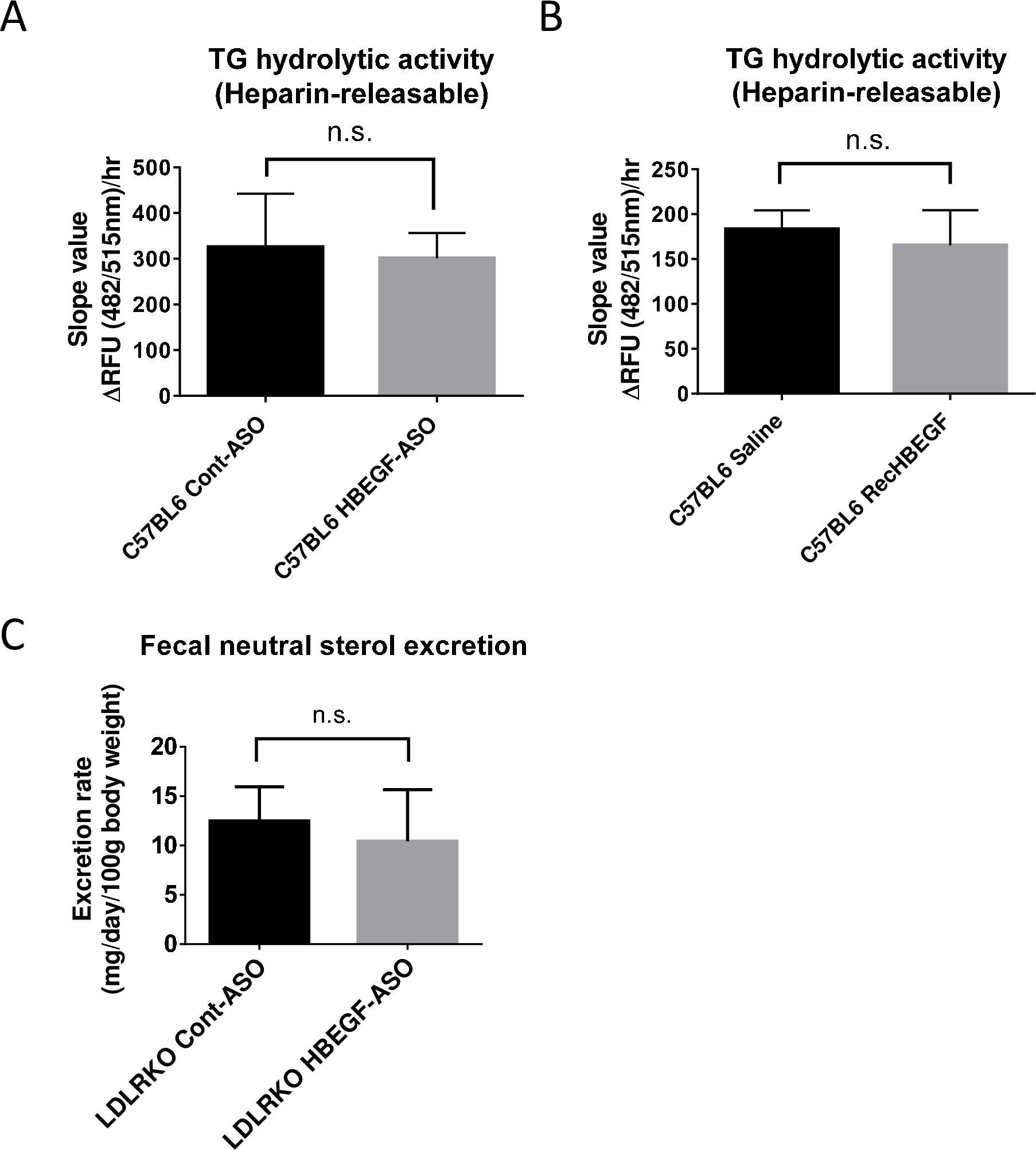

Supplement: S7 Fig — (A) Heparin-releasable plasma TG hydrolytic activities were measured in C57BL/6 mice, which is genetic background of LDLR KO mice, after 3 weeks of control or HB-EGF ASO administrations (50 mg/kg/wk) (N = 5). Downregulation of hepatic HB-EGF expression levels by the HB-EGF ASO administration was separately confirmed by qRT-PCR. (B) Heparin-releasable plasma TG hydrolytic activities were measured in C57BL/6 mice (male, 10 weeks of age) after one time tail-vein injection of either saline or recombinant HB-EGF (2 mg/kg of body weight; human active form) at 2 hours before heparin injection. (N = 5) (C). The HB-EGF ASO administration for 6 weeks (40 and 20 mg/kg/wk for 4 and 2 weeks consequently) in LDLR deficient mice under normal diet did not change fecal neutral sterol excretion rate. (N = 5) Refer to Supplemental Procedure-Extended for the procedure details. n.s. = not significant. (TIF) [file pone.0182566.s007.tif]
