# Supplementary material for: Targeting hepatic heparin-binding EGF-like growth factor (HB-EGF) induces anti-hyperlipidemia leading to reduction of angiotensin II-induced aneurysm development
Source: PLoS One. 2017 Aug 9;12(8):e0182566. doi: 10.1371/journal.pone.0182566 (PMC5549937; doi:10.1371/journal.pone.0182566)
Supplement: S1 Table — (PDF) [file pone.0182566.s008.pdf]

S1 Table. Primer sequence information used for PCR reactions.

Application	Target	Direction	Primer sequence (5'→3')
qRT-PCR	GAPDH	Forward	GGA GAA ACC TGC CAA GTA TGA
		Reverse	TCC TCA GTG TAG CCC AAG A
qRT-PCR	HB-EGF (Set1)	Forward	GCC TCC TGT AAT TGC TCT GTT A
		Reverse	CTC ACT CGA TCC TGC TTT CTT C
qRT-PCR	HB-EGF (Set2)	Forward	CTG CCA AAT CCC AGA AGA GAT
		Reverse	CCC GAA GAA CAG CAG GAT AAG
qRT-PCR	HB-EGF (Set3)	Forward	GGG AGC TTT GGA GTT GAC TAT T
		Reverse	TGA GAC CGT AGT CTC CTT ACC
PCR	GAPDH	Forward	GGA GAA ACC TGC CAA GTA TGA
		Reverse	CCT GTT GCT GTA GCC GTA TT
PCR	HB-EGF	Forward	GAT GTC CCT GGA GCA GAA TAT C
		Reverse	CAG TTC AGA CAT GGC ACT AGA A
PCR	Betacellulin	Forward	CTG GTG GTC TGC TTG ATA GTG
		Reverse	GTT TGG TTG GTT GGT TGG TTA G
PCR	EGF	Forward	GGG CTA TCC CAT CGG TAA TAA G
		Reverse	GGG ACC ACA GTC ATC TTC ATA G
PCR	Epigen	Forward	GTT GTC ACT GCA GGA AGA TTT G
		Reverse	TTC GTG GGT ATT TGA GGG TTA G
PCR	Epiregulin	Forward	GGA ATC TCC CTG GAA CAC ATA C
		Reverse	CTG GAG ACT GGA AAC AGG TTA G
PCR	Neuregulin	Forward	CGT CAT CTC CAG TGA GCA TAT T
		Reverse	GCC CAG GAA TGG TGT ATC TT
PCR	TGF-α	Forward	CAC TGG CCC TCT CTT GAT ATT T
		Reverse	GTC AGA GTG TTG CTG GTC TAT C
PCR	Amphiregulin (Set1)	Forward	GGA AGA GAG GTT TCC ACC ATA A
		Reverse	CAC ACC GTT CAC CAA AGT AAT C
PCR	Amphiregulin (Set2)	Forward	GGT TTC CAC CAT AAG CGA AAT G
		Reverse	GGA TGA TGG CAG AGA CAA AGA
PCR	EGFR	Forward	ACC ACG TCT GCA ATC CTT TAT
		Reverse	GGA AGA AAC TGG AAG GTG AGA G
PCR	ERBB4	Forward	TCA CCA AAC TGC TGC TAC TC
		Reverse	TCC CAC TAG CTC CCT CTA TTC
