# Supplementary material for: Targeting hepatic heparin-binding EGF-like growth factor (HB-EGF) induces anti-hyperlipidemia leading to reduction of angiotensin II-induced aneurysm development
Source: PLoS One. 2017 Aug 9;12(8):e0182566. doi: 10.1371/journal.pone.0182566 (PMC5549937; doi:10.1371/journal.pone.0182566)
Supplement: S1 File — (PDF) [file pone.0182566.s009.pdf]

S1 File.

Materials and Methods-Extended

Materials and reagents: Control ASO (549144: 5′- GGCCAATACGCCGTCA -3′) and HB-EGF ASO (597622: 5 ′-TACATTATAGTCTTGG -3′) were synthesized and purified by Ionis as previously described [1].The underlined text indicate cEt modified bases [2]. Poloxamer-407, a lipoprotein lipase inhibitor, was purchased from Sigma-Aldrich (Cat No. 16758). Recombinant HB-EGF (human) was purchased from R&D systems (Cat No. 295-HE-CF).

Animals: Male LDLR deficient mice (colony bred from original stock from The Jackson Laboratory; Stock No. 002207; 16 weeks of age; The strain has been backcrossed to C57BL/6J mice for 10 generations) were used for study. The mice were treated with either control or HB-EGF ASOs at a dose of 40 mg/kg/wk for 10 weeks. To induce hyperlipidemia in the mice, HFD (Harlan, Cat No TD-88137) was fed ad libitum during the last 5 weeks. To expedite lesion and aneurysm development, AngII infusion (1,000 ng/kg/min, Bachem, Torrance, CA),) via osmotic minipump implanted during the last 4 weeks of the study.

All mice were maintained in an American Association for Accreditation of Laboratory Animal Care (AAALAC) approved animal facility under protocols approved by the Institutional Animal Care and Use Committee (IACUC) of the University of Kentucky. Topically analgesic application was performed immediately after and 3-4 hours post-surgery to ameliorate pain at incision site.
http://dx.doi.org/10.17504/protocols.io.iyacfse

Noninvasive tail cuff method to measure blood pressure: Blood pressure is measured using the Kent blood pressure machine as described in the previous report [3]. This unit is a computerized, non−invasive tail−cuff system for measuring mouse systolic and diastolic blood pressure. It will simultaneously

measure blood pressure on eight mice. A platform is warmed to 37 ^{o}C to keep mice warm. Mice are placed in plexi−glass holders. Tails are threaded through an O−ring and VPR−ring. The 10 preliminary and 10 actual measurements are made in 15 minutes. The mice are housed comfortably in holders. We measured blood pressure three consecutive days at the same time. Only mice that measured 50% (10 measurements) with a SD < 30 mmHg were accepted. http://dx.doi.org/10.17504/protocols.io.iygcftw

In situ quantification of aneurysm severity and atherosclerotic lesion size - Mice were perfused with saline, and hearts with attached aortas were harvested. Aortas were placed in 10% neutral buffered formalin overnight and then transferred to phosphate buffered saline (PBS). After removal of adventitia, aortas were photographed using a digital camera (DS-Ri1; Nikon Instruments) to later measure the maximal diameter of the abdominal ascending aorta. Then, the aortas were cut open longitudinally, pinned, and photographed *en face* with mm unit ruler for size reference. Areas of thoracic ascending aortic intima and atherosclerotic lesions in the aortic arch area were quantified using Image Pro 7.0 software (Media Cybernetics, Bethesda, MD) as described previously [4,5]. http://dx.doi.org/10.17504/protocols.io.ix7cfrn; http://dx.doi.org/10.17504/protocols.io.izicf4e; http://dx.doi.org/10.17504/protocols.io.izfcf3n

Lipoprotein cholesterol distribution assay by FPLC: Blood was collected from mice in EDTA coated tubes by cardiac puncture and plasma was isolated by centrifugation. The cholesterol distribution among lipoprotein classes was determined after separation of plasma by gel filtration chromatography based upon the method described previously [6]. Briefly, an aliquot of plasma was diluted to 0.5 µg total cholesterol/µL in 0.9% NaCl, 0.05% EDTA/NaN_3 and centrifuged at 2000xg for 10 minutes to remove any particulate debris. The supernatant was transferred to a glass insert contained in a GC vial. After loading the vial into an autosampler set at 4°C (Agilent Technologies, G1329A), 40 µL of sample was injected onto a Superose 6 10/300 (GE Healthcare Life Sciences) chromatography column. Under control

of an isocratic pump (Agilent Technologies, G1310A/B), the sample was separated at a flow rate of 0.4 ml/min with eluent containing 0.9% NaCl, 0.05% EDTA/NaN_3. Column effluent was mixed with total cholesterol enzymatic reagent (Pointe Scientific) running at a flow rate of 0.125 mL/min and the mixture was passed through a knitted reaction coil (Aura Industries Inc., EPOCOD) in a 37°C H_2O jacket. The absorbance of the reaction mixture was read at 500 nm using a variable wavelength detector (Agilent Technologies, G1314F). The signal was subsequently integrated using Agilent Open LAB Software Suite (Agilent Technologies). VLDL, LDL, and HDL cholesterol concentrations were calculated by multiplying the total plasma cholesterol concentration by the cholesterol percentage within the elution region for each lipoprotein class. http://dx.doi.org/10.17504/protocols.io.izhcf36

Quantification of liver tissue lipid content: We followed procedure described by Temel RE et al. [7]. Approximately 50 mg of frozen liver was thawed, minced and placed into a 16x100mm glass tube with a Teflon-lined cap. Lipids were extracted with 3 ml of 2:1 chloroform:methanol ($CHCl_3$:MeOH) overnight at room temperature. The lipid extract was then transferred to a new 16x100 mm glass screw top tube following centrifugation at 1,500xg for 10 min. The lipid extract was dried down under nitrogen while being heated at 55°C. The dried lipid extract was dissolved in 6ml of 2:1 $CHCl_3$:MeOH with the addition of 1.2ml dilute H_2SO_4 (0.05%, v/v) for phase separation. The upper aqueous phase was removed after vigorous vortexing and centrifuged as described above. An aliquot of the bottom organic phase was mixed with 2ml 1% Trition-X100 dissolved in $CHCl_3$. The samples were dried down under nitrogen and re-dissolved in 1 ml water while being heated 60°C for 10 min. After centrifugation at 1,500xg for 5 min, the samples were analyzed for lipids using commercially available enzymatic kits for TG, total and free cholesterol (Wako diagnostics). The Lowry assay was used to determine protein content of the lipid-extracted liver that had been dried and then dissolved in 1N NaOH.
http://dx.doi.org/10.17504/protocols.io.iy7cfzn

Hepatic VLDL secretion assay: The procedure described by Willecke F. *et al.* was employed for the assay [8]. Standard C57BL/6 mice (male, 8-10weeks of age) from the Jackson Laboratory (Stock No 005061) were fasted for 4 hours before initiation of VLDL secretion assay. After baseline blood collection (~50ul) from the retro-orbital sinus, mice were intraperitoneally injected with poloxamer-407 (P-407) (10% (w/v) in saline, Sigma-Aldrich, Cat No 16758) at a dose of 1.0 g/kg of body weight. After 1, 2 and 5 hours, approximately 50ul of blood was collected from the retro-orbital sinus. Blood collection and injections were conducted on mice sedated under isoflurane (inhalation with 1-4% in O_2). TG and apoB concentrations, as surrogates of VLDL, in plasma samples were measured by enzymatic quantification assay kits purchased from Wako Diagnostics and Western blotting, respectively. http://dx.doi.org/10.17504/protocols.io.izxcf7n

Heparin-releasable lipoprotein lipase (LPL) activity assay: The procedure described by Willecke F. et al. was followed with modification of heparin doses and bleeding times [8]. Male C57BL/6 mice were fasted for 4 hrs and heparin dissolved in saline (1.5 U/g of body weight, Sigma, H3393) was injected via tail vein (N=5 per group). Approximately 50 ul of blood was collected at 0 and 20 min time points after heparin injection via retro-orbital route under isoflurane (inhalation with 1-4% in O_2) . Total TG hydrolytic activities of plasma samples were measured using a fluorogenic assay kit purchased from Abcam (ab204721) following the manufacturer's recommendations.
http://dx.doi.org/10.17504/protocols.io.iy3cfyn

GC analysis of fecal neutral sterols: We followed procedure described by Temel RE et al. [7]. Mice were singly housed in wire bottom cages, fed *ad libitum,* and feces were collected for 72 hours. Feces were transferred to a 20mL glass scintillation vial and desiccated overnight in a vacuum oven set at 80°C. The dried feces were weighed and crushed into a fine powder using a mortar and pestle. A portion of fecal powder (~50 mg) was added to a 16x100 mm glass screw top tube containing 0.2 mg 5-alpha cholestane (Steraloids, C3300-000). To saponify fecal lipid, 2 ml 95% EtOH and 200 μL 50% KOH was added to

the tube which was then sealed with a Teflon-lined cap and incubated at 60°C for 3 hours with periodic vortexing. Neutral sterol was extracted from the sample by adding 2 mL hexane followed by 2 mL H_2O with vigorous vortexing between each addition. The tube was centrifuged at 1500xg for 10 min at room temperature. The upper hexane phase (~0.5 ml) was transferred to a GC vial for analysis of sterol mass. The extracted sterol was analyzed by injecting 1µL of sample into a ZB50 (0.53-mm inner diameter × 15 m × 1 µm) gas-liquid chromatography column (Phenomenex) at 250°C installed on a Agilent Technologies 7890B gas chromatograph equipped with a Agilent Technologies 7693 autosampler using on-column injection and a flame ionization detector. The µg of cholesterol and coprostanol in the sample was then calculated using the following equation:

$$mg\ sterol = \frac{peak\ area\ sterol}{peak\ area\ 5 \propto cholestane} \times \frac{1}{sterol\ RRF} \times 0.2\, mg\ 5 \propto cholestane$$

Relative response factor (RFF) was 0.76 for cholesterol and 0.92 for coprostanol. Fecal neutral sterol excretion expressed as mg/day/100 g body weight and was calculated using the following equation:

$$(mg\ cholesteorl + coprostanol) \times \frac{mg\ feces\ collected}{mg\ feces\ analyzed} \times \frac{1}{3\ days} \times \frac{100}{g\ body\ weight}$$

http://dx.doi.org/10.17504/protocols.io.iy9cfz6

Quantitative RT-PCR: Total RNAs were extracted from mouse tissues using an RNA isolation kit (Qiagen, Cat No. 74106) following the manufacturer's recommendations. cDNAs were prepared using cDNA synthesis kits (Bio-Rad iScript™ cDNA Synthesis Kit, Cat No. 1708891). For amplification and quantification of PCR products, Maxima SYBR Green/ROX qPCR Master Mix (2X) (ThermoFisher Scientific; Cat No. K0223) and a PCR amplification apparatus (Bio-Rad, CFX96 Touch™ Real-Time PCR System) were used. Expression level of GAPDH mRNA was used for normalization. More than two

sets of primers were tested for optimization of PCR amplification for each gene. Primer sequence information is described in **Supplementary Table 1**.

Western Blotting: Freshly isolated liver samples were lysed in RIPA buffer (Cell Signaling; Cat No. 9806) containing PMSF (1mM) and protease and phosphatase inhibitor cocktails (Sigma-Aldrich; Cat No. P8340 and P5726). Protein samples were separated by SDS-PAGE using 4-20% Bio-Rad Mini-PROTEAN TGX gels following a standard procedure. Separated proteins were transferred onto polyvinylidene difluoride (PVDF) membranes using a Trans-Blot® Turbo™ Transfer System (Bio Rad, Cat No. 1704155). After incubation with an anti-apoB antibody (HRP-conjugated; Meridian Life Science, Cat No. K34005G-1) in 1% BSA or 5% fat free milk containing TBST solution, apoB bands were detected using enhanced chemiluminescent prime solution (ECL; Amersham, Cat No. RPN2232). ApoB bands were captured and quantified using image analysis (myECL image, Thermo Fisher, Cat No. 62236).

Statistical analysis: Results are presented as mean ± SD. Test group samples are compared to control group samples by Student's t-test or by Two-way ANOVA for time-dependent lipid level changes. If required, multiple comparison correction by the Benjamini Hochberg method was applied. A significant p value less than 0.05 was considered statistically different.

References for procedure:

1. Seth PP, Vasquez G, Allerson CA, Berdeja A, Gaus H, et al. (2010) Synthesis and biophysical evaluation of 2',4'-constrained 2'O-methoxyethyl and 2',4'-constrained 2'O-ethyl nucleic acid analogues. J Org Chem 75: 1569-1581.
2. Lee RG, Fu W, Graham MJ, Mullick AE, Sipe D, et al. (2013) Comparison of the pharmacological profiles of murine antisense oligonucleotides targeting apolipoprotein B and microsomal triglyceride transfer protein. J Lipid Res 54: 602-614.

3. Rateri DL, Moorleghen JJ, Balakrishnan A, Owens AP, 3rd, Howatt DA, et al. (2011) Endothelial cell-specific deficiency of Ang II type 1a receptors attenuates Ang II-induced ascending aortic aneurysms in LDL receptor-/- mice. Circ Res 108: 574-581.

4. Daugherty A, Whitman SC (2003) Quantification of atherosclerosis in mice. Methods Mol Biol 209: 293-309.

5. King VL, Szilvassy SJ, Daugherty A (2002) Interleukin-4 deficiency decreases atherosclerotic lesion formation in a site-specific manner in female LDL receptor-/- mice. Arterioscler Thromb Vasc Biol 22: 456-461.

6. Kieft KA, Bocan TM, Krause BR (1991) Rapid on-line determination of cholesterol distribution among plasma lipoproteins after high-performance gel filtration chromatography. J Lipid Res 32: 859-866.

7. Temel RE, Lee RG, Kelley KL, Davis MA, Shah R, et al. (2005) Intestinal cholesterol absorption is substantially reduced in mice deficient in both ABCA1 and ACAT2. J Lipid Res 46: 2423-2431.

8. Willecke F, Scerbo D, Nagareddy P, Obunike JC, Barrett TJ, et al. (2015) Lipolysis, and not hepatic lipogenesis, is the primary modulator of triglyceride levels in streptozotocin-induced diabetic mice. Arterioscler Thromb Vasc Biol 35: 102-110.
